# Fatigue-induced alterations in arm swing contribution to countermovement jump mechanics

**DOI:** 10.1186/s13102-026-01650-8

**Published:** 2026-03-12

**Authors:** Recep Soslu, Meriç Eraslan, Murat Taş, Meltem Devrilmez, İzzet Kırkaya, Ali Özkan, Hakan Yarar, İsmail Can Çuvalcıoğlu, Ali Ahmet Doğan, Abdullah Uysal, Sabri Gerger

**Affiliations:** 1 Faculty of Sports Sciences, Karamanoğlu Mehmetbey University, Karaman, Türkiye; 2 Faculty of Sports Sciences, Akdeniz University, Antalya, Türkiye; 3 Faculty of Sports Sciences, Manisa Celal Bayar University, Manisa, Türkiye; 4 Institute of Health Sciences, Karamanoğlu Mehmetbey University, Karaman, Türkiye; 5 Faculty of Sports Sciences, Bozok University, Yozgat, Türkiye; 6 Faculty of Sports Sciences, Kırıkkale University, Kırıkkale, Türkiye

**Keywords:** Countermovement jump, Arm swing, Neuromuscular fatigue, Bayesian analysis, Force–power, Phase-specific acceleration

## Abstract

**Purpose:**

This study examined protocol-specific neuromuscular responses to acute fatigue during countermovement jump (CMJ) performance by comparing jumps performed with arm swing (CMJ_AS_) and without arm swing (CMJ_NAS_).

**Methods:**

Eighteen physically active male participants (age: 21.4 ± 1.4 years; height: 188.2 ± 5.7 cm; body mass: 80.5 ± 5.4 kg) performed CMJ with and without arm swing on separate days before and after acute fatigue induced by a 30-s Wingate test. Ground reaction forces were collected via force platform, and biomechanical variables were analyzed using Bayesian paired-samples t-tests and repeated-measures ANOVA.

**Results:**

Bayesian analyses revealed extreme evidence for pre–post changes in vertical take-off velocity in both CMJ_AS_ (BF₁₀=369.74) and CMJ_NAS_ (BF₁₀=40.45), accompanied by extreme time×condition interaction evidence (BF_incl_=6142.38), indicating protocol-dependent modulation of take-off mechanics. Jump height derived from flight time showed extreme evidence in CMJ_AS_ (BF₁₀=219.89) and very strong evidence in CMJ_NAS_ (BF₁₀=17.48). CMJ_NAS_ demonstrated stronger evidence for fatigue-related alterations in relative maximal power (BF₁₀=20.05), peak concentric force (BF₁₀=2.49), and late push-off acceleration (BF₁₀=12.86), whereas corresponding changes in CMJ_AS_ were moderate or anecdotal. In contrast, average force, average power, early push-off acceleration, and eccentric braking variables showed little to no evidence for change across conditions.

**Conclusion:**

CMJ without arm swing is more sensitive to fatigue-induced alterations in lower-limb neuromuscular function, particularly in force–power output and late-phase propulsion. In contrast, CMJ with arm swing preserves global performance through whole-body coordination. These findings underscore the importance of protocol selection when CMJ is used for neuromuscular monitoring and fatigue assessment.

## Introduction

The countermovement jump (CMJ) is one of the most frequently used biomechanical tasks for evaluating neuromuscular performance in both individual and team sport athletes [1]. CMJ performance reflects the integrated contribution of force production, velocity generation, and intersegmental coordination, and is therefore widely applied as a practical indicator of lower-extremity neuromuscular function. The mechanical basis of CMJ performance is primarily governed by the efficiency of the stretch–shortening cycle (SSC), which enables elastic energy storage during the eccentric phase and its reutilization during the subsequent concentric phase, thereby enhancing force output and mechanical efficiency [2, 3]. Biomechanically, the CMJ consists of a downward eccentric braking phase followed by a rapid concentric propulsion phase that culminates in take-off [4, 5].

Acute neuromuscular fatigue has been shown to disrupt SSC efficiency by impairing motor unit firing behavior, reducing force–velocity coupling, and altering the temporal structure of force application [6–8]. High-intensity anaerobic exercise, such as the Wingate anaerobic test, represents a well-established model for inducing acute peripheral and central fatigue, which can negatively affect jump performance and movement coordination [9, 10]. Previous studies have demonstrated that fatigue may prolong eccentric and concentric phase durations, reduce maximal concentric velocity, and modify force–time and velocity–time curves, even in cases where jump height remains relatively unchanged [6, 11, 12]. These findings suggest that fatigue-related neuromuscular impairments may not always be adequately captured by global performance outcomes alone.

Importantly, the manifestation of fatigue-induced alterations in CMJ performance is not uniform and may depend on the specific jump protocol employed. CMJ is commonly performed either with unrestricted arm swing (CMJ_AS_) or with the hands positioned on the hips (CMJ_NAS_) [1, 4]. Allowing arm swing enhances jump performance by increasing vertical impulse and facilitating momentum transfer from the upper to the lower extremities, thereby augmenting take-off velocity and jump height [13, 14]. While this intersegmental contribution improves overall mechanical output, it may also obscure fatigue-related deficits in lower-limb neuromuscular function by introducing compensatory upper-body strategies, particularly under conditions of acute metabolic stress [6, 12]. In contrast, CMJ_NAS_ minimizes upper-extremity involvement and reduces intersegmental compensation, providing a more isolated representation of lower-limb force–velocity behavior [4, 15]. As a result, CMJ_NAS_ has been proposed as a more sensitive protocol for detecting acute fatigue-induced alterations in neuromuscular performance following high-intensity tasks such as the Wingate anaerobic test [6, 12]. From both research and applied perspectives, distinguishing between CMJ_AS_ and CMJ_NAS_ is therefore critical, as protocol selection may substantially influence the interpretation of fatigue-related changes in CMJ-derived variables.

Beyond jump height, CMJ performance can be characterized using a wide range of kinetic and kinematic variables derived from force platform data, offering deeper insight into neuromuscular strategy under fatigue [15–16]. Although jump height calculated from take-off velocity (JHTOV) and flight time (JHFT) remains a commonly reported outcome, these global metrics may fail to capture subtle fatigue-induced adaptations in force application and movement timing [12, 17]. Force- and acceleration-based variables (including relative maximal power (RMP), peak concentric force (FPC), average power (AP), and phase-specific accelerations during the push-off phase (AFPO and ASPO)) provide more detailed information regarding how force is generated, timed, and redistributed across movement phases under fatigue [3, 5, 18]. Additionally, eccentric braking metrics such as force at peak eccentric (FPE) and eccentric deceleration phase impulse (EDP) offer valuable insight into SSC preservation and the capacity to absorb and reutilize mechanical energy following acute fatigue [3, 19, 20]. Previous research suggests that eccentric force characteristics may be partially preserved during early stages of fatigue or compensated through altered movement strategies, highlighting the importance of phase-specific analysis rather than reliance on jump height alone [6, 21]. Collectively, examining CMJ variables within a system-based framework enables a more comprehensive understanding of fatigue-related biomechanical adaptations and allows clearer differentiation between true neuromuscular fatigue and compensatory strategies arising from arm swing involvement.

Despite the widespread use of CMJ for fatigue monitoring, there remains limited evidence directly comparing how acute anaerobic fatigue differentially affects force–velocity and phase-specific CMJ variables under arm swing and no-arm-swing conditions. Therefore, the purpose of the present study was to investigate the effects of acute fatigue induced by a Wingate anaerobic test on a comprehensive set of CMJ kinetic and kinematic variables, comparing CMJ_AS_ and CMJ_NAS_ protocols. By adopting a system-based analytical approach, this study aims to provide a more mechanistic understanding of fatigue-related alterations in vertical jump performance.

## Methods

### Participants and ethical approval

Eighteen recreationally active male university students voluntarily participated in this study. Participant characteristics were as follows: age 21.43 ± 1.40 years, height 188.18 ± 5.70 cm, body mass 80.50 ± 5.40 kg, and body mass index (BMI) 22.60 ± 1.20 kg·m⁻². Physical activity level was assessed using the International Physical Activity Questionnaire–Short Form (IPAQ-SF), and weekly physical activity was calculated as metabolic equivalent task (MET) minutes per week. All participants demonstrated a moderate level of habitual physical activity (865.56 ± 68.54 MET·min·week⁻¹), indicating a relatively homogeneous recreationally active cohort [22]. Participants were eligible for inclusion if they were male university students aged 18–25 years, engaged in regular recreational physical activity without participation in structured competitive training programs, free from lower-limb musculoskeletal injury within the previous six months, and without known neurological, cardiovascular, or metabolic disorders that could contraindicate maximal anaerobic exercise or explosive jumping tasks. Individuals were excluded if they reported current pain or injury affecting the lower extremities, trunk, or upper limbs; had undergone lower-limb surgery within the previous year; were using medications or supplements known to influence neuromuscular function or fatigue responses; or failed to comply with pre-test instructions, including refraining from strenuous physical activity prior to testing sessions. An a priori power analysis conducted using G*Power (v3.1) indicated that a minimum sample size of 12 participants was required to detect a large effect size (η²=0.40) with an alpha level of 0.05 and statistical power of 0.80, and the final sample size of 18 participants exceeded this requirement. Initially, a total of 18 athletes were assessed for eligibility. After applying the predefined inclusion and exclusion criteria, 18 participants met al.l requirements and were included in the final analysis. This study was conducted in accordance with the Declaration of Helsinki. Ethical approval was obtained from the [Non-Invasive Clinical Research Ethics Committee of Karamanoğlu Mehmetbey University] (Approval No: 26793/2021). Written informed consent to participate was obtained from all participants prior to data collection. Participation was voluntary, and all participants were informed that they could withdraw from the study at any time without any consequences.

### Study design

The study was conducted using a repeated-measures, within-subject experimental design. Participants completed CMJ assessments under two conditions: CMJ_AS_ and CMJ_NAS_. Measurements were obtained before and after the application of a Wingate anaerobic test (WAnT) to evaluate acute changes in CMJ biomechanics. Each participant performed all experimental conditions, and all measurements were collected following a standardized testing protocol. Testing sessions were distributed across three separate days. All assessments were performed at the same time of day for each participant. The order of CMJ conditions was determined using a randomized block sequence. Participants were instructed to follow the same preparation procedures prior to each session, including maintaining consistent footwear and habitual dietary intake and avoiding strenuous physical activity before testing. This design allowed for systematic comparison of CMJ performance across conditions and time points.

### Fatigue induction protocol

Acute neuromuscular fatigue was induced using a standardized 30-s Wingate anaerobic test (WAnT) performed on a mechanically braked cycle ergometer (Lode Excalibur Sport, Groningen, Netherlands). Resistance was set at 0.75 N·m·kg⁻¹ body mass. Following a 5-min unloaded warm-up and a 2-min passive recovery, participants performed a 30-s all-out sprint against the prescribed resistance. Strong verbal encouragement was provided throughout the test to ensure maximal effort. To minimize the influence of post-activation potentiation and capture acute fatigue effects, post-fatigue CMJ testing commenced immediately (within ~ 30 s) following completion of the Wingate test. This time window has been shown to favor fatigue-related performance decrements over potentiation effects following high-intensity anaerobic exercise [23].

### Countermovement jump testing and outcome measures

All CMJ trials were performed on a three-dimensional force platform (Kistler 5691 A; 600 × 500 × 50 mm; Kistler, Winterthur, Switzerland) firmly mounted on the laboratory floor. Vertical ground reaction forces were sampled at 1,000 Hz and processed using proprietary software (Kistler Measurement, Analysis and Reporting Software; MARS). Gravitational acceleration was applied to derive kinetic and kinematic variables from force–time data. Prior to each trial, participants were instructed to stand still to ensure stable baseline force signals, and identical verbal instructions and encouragement were provided throughout testing to ensure maximal and consistent effort. CMJ performance was assessed under two experimental conditions: CMJ_AS_ and CMJN_AS_. For CMJ_AS_, participants began from an upright standing position with feet positioned shoulder-width apart and arms free to move naturally. After confirming even weight distribution on the force platform, participants performed a self-selected countermovement followed by a maximal vertical jump with unrestricted arm swing, landing in an athletic position on the platform. For CMJ_NAS_, participants adopted the same stance but maintained their hands on their hips throughout the movement to minimize upper-limb contribution. Visual feedback was provided prior to movement initiation to ensure symmetrical weight distribution. In both protocols, participants selected their own countermovement depth to reflect natural jumping mechanics. For each CMJ condition, three maximal trials were performed with standardized rest intervals of approximately (60–90 s) between attempts. This duration was selected based on commonly used vertical jump testing protocols, which indicate that rest intervals within this range are sufficient to minimize fatigue and maintain maximal performance across repeated trials. Trials were repeated if technical criteria were violated, including removal of hands from the hips during CMJ_NAS_, excessive knee flexion during flight or landing, or loss of balance upon ground contact. The best trial for each condition, based on jump height, was retained for subsequent analysis, consistent with established CMJ assessment guidelines.

CMJ performance was quantified using a comprehensive set of kinetic and kinematic variables derived from force-platform data. To facilitate biomechanical interpretation and reduce redundancy, outcome measures were grouped into functional domains reflecting distinct aspects of neuromuscular performance. Global performance outcomes included jump height calculated from take-off velocity (JHTOV, m), jump height calculated from flight time (JHFT, m), vertical take-off velocity (VTOV, m·s⁻¹), and total flight time (FT, s), representing the overall mechanical output of the jump. Neuromuscular force–power production was characterized using relative maximal power (RMP, W·kg⁻¹), peak concentric force (FPC, N·kg⁻¹), average power (AP, W), and average force (AF, N), providing insight into the capacity of the lower limbs to generate force and power under maximal effort conditions. Phase-specific acceleration and coordination were assessed using mean acceleration (A, m·s⁻²), acceleration during the first half of the push-off phase (AFPO, m·s⁻²), acceleration during the second half of the push-off phase (ASPO, m·s⁻²), average velocity (AV, m·s⁻¹), and time to maximal force (TMF, s), allowing examination of temporal and strategic adaptations in force application under fatigue. Finally, eccentric braking and stretch–shortening cycle (SSC) preservation were evaluated using eccentric deceleration phase impulse (EDP, N·s) and force at peak eccentric (FPE, N·kg⁻¹), which reflect the ability to absorb and reutilize mechanical energy during the eccentric phase of the CMJ.

### Timing of countermovement jump assessments

CMJ assessments were conducted at two predefined time points relative to the fatigue protocol: pre-fatigue and post-fatigue. CMJ_AS_ and CMJ_NAS_ were assessed on separate testing days, with each testing day dedicated to a single CMJ condition. At the beginning of each testing day, participants first completed the familiarization procedures (if applicable), followed by a standardized warm-up protocol consisting of dynamic stretching, low-to-moderate intensity locomotor activities, and submaximal jumping tasks. After completion of the warm-up, pre-fatigue CMJ measurements were performed for the assigned condition according to the predefined randomized testing schedule. A standardized rest interval was provided between successive CMJ trials to allow participants to return to a resting standing position before initiating the next attempt.

**Fig. 1:**
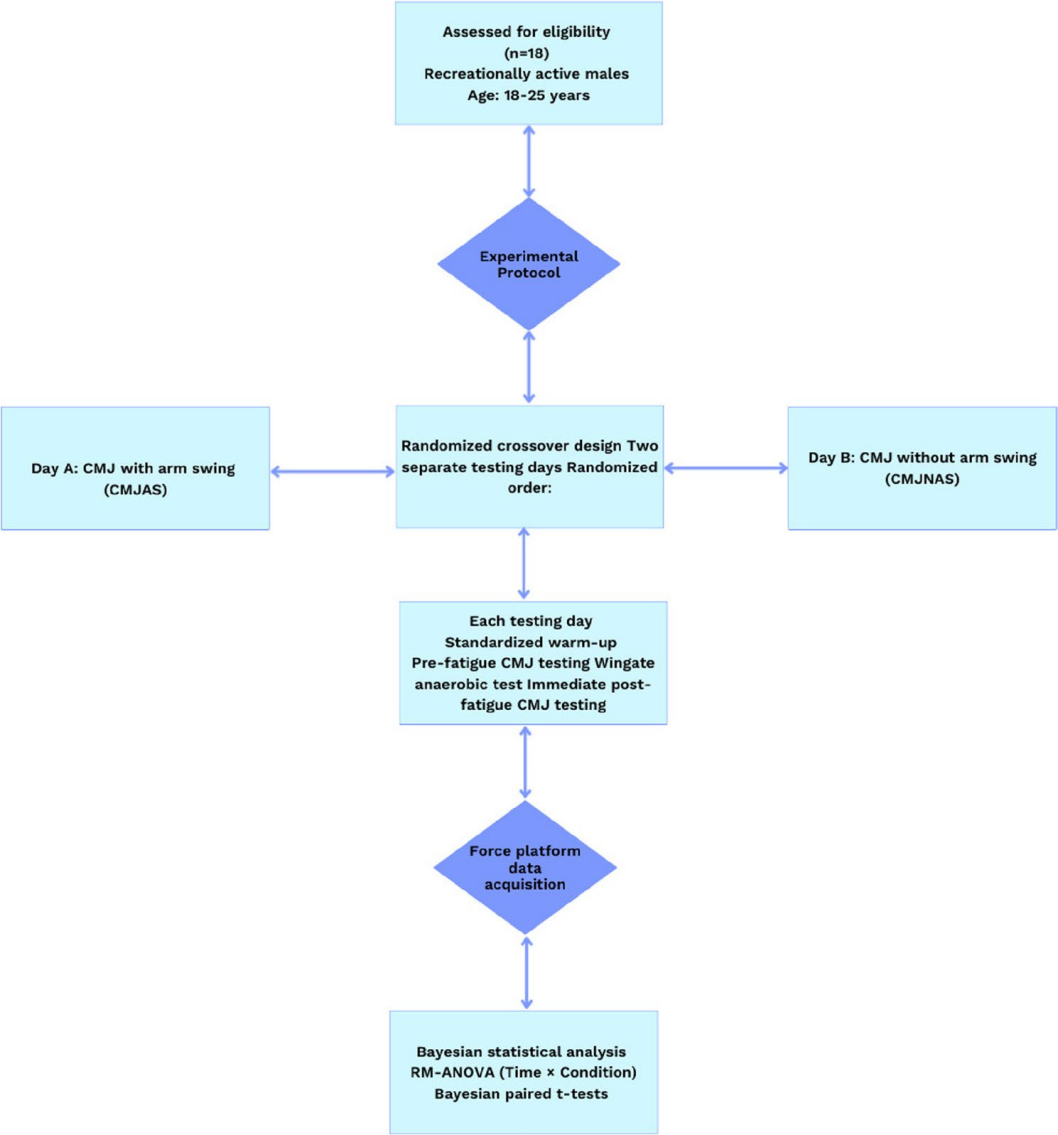
Flow diagram for the experimental procedure of the study

Immediately after completion of the pre-fatigue CMJ assessments, participants proceeded to the fatigue induction protocol. Neuromuscular fatigue was induced using the Wingate anaerobic test (WAnT), as described above. Upon completion of the Wingate test, participants transitioned directly from the cycle ergometer to the force platform area. Post-fatigue CMJ assessments for the same jump condition were initiated immediately following the fatigue protocol, with no additional exercise performed between the Wingate test and the CMJ trials. Post-fatigue CMJ testing was conducted using the same warm-up status, procedures, instructions, and execution criteria as those applied during pre-fatigue assessments. Participants completed post-fatigue CMJ trials for the same condition tested earlier that day, following the predefined randomized order of testing days. The number of trials, rest intervals between jumps, and acceptance criteria for valid trials were kept identical between pre-fatigue and post-fatigue conditions to ensure temporal and procedural consistency across all measurements (Fig. 1).

### Statistical analysis

All statistical analyses were conducted using a Bayesian inference framework. Bayesian repeated-measures analysis of variance (ANOVA) was applied separately to each dependent variable with two within-subject factors: time (pre-fatigue vs. post-fatigue) and condition (CMJ_AS_ vs. CMJ_NAS_). The analyzed outcome variables included performance measures: JHTOV, JHFT, VTOV, and FT; neuromuscular force–power variables: RMP, FPC, AP, and AF; phase-specific acceleration and coordination variables: mean acceleration (A), AFPO, ASPO, AV, and TMF; and eccentric braking and SSC related variables: EDP and FPE. Bayes Factors for inclusion (BF_incl_) were calculated to quantify the evidence supporting the inclusion of main effects and interaction terms for each outcome variable. Evidence strength was interpreted using established thresholds, whereby BF_incl_ values between 1 and 3 indicated anecdotal evidence, 3 to 10 moderate evidence, 10 to 30 strong evidence, 30 to 100 very strong evidence, and values greater than 100 extreme evidence in favor of effect inclusion; BF_incl_ values below 1 were interpreted as evidence favoring exclusion of the effect. In accordance with factorial analysis principles, interaction effects (time × condition) were examined prior to the interpretation of main effects. When evidence supported the presence of a time×condition interaction, planned Bayesian paired-samples t-tests were conducted to examine pre- to post-fatigue changes within each CMJ condition. In addition to Bayes Factors, posterior estimates were summarized using 95% Bayesian credible intervals (CrI) to describe uncertainty around parameter estimates. Credible intervals were interpreted as the range within which the true parameter value lies with a 95% posterior probability, conditional on the observed data and model assumptions. Bayes Factors were interpreted exclusively as measures of evidential strength and were not used as indicators of effect magnitude or practical significance. To provide additional context regarding measurement variability, coefficients of variation (CV%) were calculated for all outcome variables. For descriptive comparison with existing literature, complementary frequentist repeated-measures ANOVA results were also reported; however, all primary inferential conclusions were based on Bayesian evidence [24]. All Bayesian analyses were conducted using JASP (Version 0.18.3, University of Amsterdam).

The statistical approach was designed to provide transparent inference within a repeated-measures framework involving multiple biomechanical outcomes. Bayesian repeated-measures ANOVA was used to quantify evidence for time, condition, and interaction effects without reliance on dichotomous significance testing, with interaction terms evaluated prior to main effects. Bayes Factors were interpreted solely as measures of evidential strength, while complementary frequentist analyses were reported descriptively for comparison with existing literature. All primary inferential conclusions were based on Bayesian evidence, ensuring methodological rigor consistent with current recommendations in sports biomechanics research.

## Results

Eighteen physically active male participants completed all testing sessions. Descriptive characteristics indicated a homogeneous sample with a normoweight profile and high habitual physical activity levels. Anaerobic performance assessed via the anaerobic test demonstrated high short-term power capacity, with a mean peak power output of 937.91 ± 127.99 W and a mean power output of 592.68 ± 53.51 W, consistent with trained young adult populations (Table 1).

**Table 1:** Participant characteristics and anaerobic performance (Mean ± SD)

Variable	Mean ± SD
Sample size (n)	18
Age (years)	21.43 ± 1.40
Height (cm)	188.18 ± 5.70
Body mass (kg)	80.50 ± 5.40
Body mass index (kg·m⁻²)	22.60 ± 1.20
Physical activity level (MET·min·week⁻¹)	865.56 ± 68.54
Peak Power (W)	937.91 ± 127.99
Mean Power (W)	592.68 ± 53.51

Descriptive statistics for countermovement jump outcomes under CMJ_AS_ and CMJ_NAS_ conditions are presented in Table 2. Bayesian paired-samples t-tests revealed extreme evidence for pre–post changes in JHFT in CMJ_AS_ (BF₁₀ = 219.89) and very strong evidence in CMJ_NAS_ (BF₁₀ = 17.48). Bayesian repeated-measures ANOVA indicated extreme evidence for a time × condition interaction (BF_incl_ = 373.79), demonstrating divergent fatigue-related responses between CMJ protocols. JHTOV showed anecdotal evidence for change in CMJ_AS_ (BF₁₀ = 1.09) and strong evidence in CMJ_NAS_ (BF₁₀ = 6.61), accompanied by strong interaction evidence (BF_incl_ = 13.38). VTOV demonstrated extreme evidence for pre–post changes in both CMJ_AS_ (BF₁₀ = 369.74) and CMJ_NAS_ (BF₁₀ = 40.45), with extreme interaction evidence (BF_incl_ = 6142.38), indicating protocol-specific modulation of take-off mechanics (Table 3).

RMP exhibited moderate evidence for change in CMJ_AS_ (BF₁₀ = 3.26) and strong evidence in CMJ_NAS_ (BF₁₀ = 20.05), with strong interaction support (BF_incl_ = 11.52). FPC showed stronger evidence in CMJ_NAS_ (BF₁₀ = 2.49) compared with anecdotal evidence in CMJ_AS_ (BF₁₀ = 0.42), accompanied by moderate-to-strong interaction evidence (BF_incl_ = 6.48). In contrast, AF and AP did not demonstrate meaningful evidence for pre–post changes or interaction effects (BF₁₀ < 3; BF_incl_ < 1), indicating stability of mean-level output metrics across conditions (Tables 2 and 3).

Phase-specific acceleration analysis revealed strong evidence for changes in ASPO in CMJ_NAS_ (BF₁₀ = 12.86), whereas CMJ_AS_ showed no meaningful evidence (BF₁₀ = 0.84). Bayesian ANOVA indicated extreme interaction evidence for ASPO (BF_incl_ = 590.17), highlighting pronounced protocol-dependent fatigue adaptations in late push-off mechanics. Early AFPO did not demonstrate meaningful evidence for change in either condition (BF₁₀ < 0.35), and interaction evidence favored exclusion of this effect (BF_incl_ = 0.36). A comprehensive summary of Bayesian evidence for within-condition changes and time × condition interactions across all outcome variables is provided in Table 3. Overall, global jump performance and phase-specific acceleration variables exhibited the strongest protocol-dependent fatigue responses, whereas mean-level force and power outputs showed limited sensitivity to acute fatigue under the present experimental conditions.

**Table 2:** Descriptive statistics for CMJ outcomes (Mean ± SD)

Variable	Pre-CMJ_AS_ Mean ± SD	Post-CMJ_AS_ Mean ± SD	Pre-CMJ_NAS_ Mean ± SD	Post-CMJ_NAS_ Mean ± SD
JHFT (m)	0.43 ± 0.05	0.41 ± 0.05	0.40 ± 0.05	0.44 ± 0.06
JHTOV (m)	0.42 ± 0.04	0.41 ± 0.05	0.39 ± 0.04	0.41 ± 0.05
VTOV (m·s⁻¹)	2.85 ± 0.18	2.74 ± 0.20	2.70 ± 0.17	2.82 ± 0.22
RMP (W·kg⁻¹)	54.2 ± 6.1	50.8 ± 6.5	51.0 ± 5.8	56.1 ± 6.9
FPC (N·kg⁻¹)	25.6 ± 2.9	24.8 ± 3.1	24.9 ± 2.7	26.1 ± 3.3
AF (N)	1680 ± 210	1605 ± 225	1610 ± 205	1595 ± 240
AP (W)	4120 ± 520	3870 ± 560	3920 ± 510	4010 ± 590
AFPO (m·s⁻²)	7.6 ± 1.0	7.4 ± 1.1	7.3 ± 0.9	7.2 ± 1.2
ASPO (m·s⁻²)	11.9 ± 1.3	10.8 ± 1.4	11.1 ± 1.2	12.6 ± 1.5
EDP (N/s)	66.46 ± 23.75	56.84 ± 12.68	75.53 ± 18.97	52.77 ± 18.38

Variables include *JHFT* jump height derived from flight time, *JHTOV* Jump height derived from take-off velocity, *VTOV* Vertical take-off velocity, *RMP* Relative maximal power, *FPC* Peak concentric force, *AF* Average force, *AP *Average power, *AFPO* Acceleration during the first and second *ASPO* Halves of the push-off phase

**Table 3:** Bayesian Evidence for Pre–Post Changes and Interaction Effects

Variable	CMJ_AS_ BF₁₀	CMJ_NAS_ BF₁₀	BF_incl_ (Time×Condition)	Evidence Summary
JHFT (m)	219.89	17.48	373.79	Extreme interaction
JHTOV (m)	1.09	6.61	13.38	Strong interaction
VTOV (m·s⁻¹)	369.74	40.45	6142.38	Extreme interaction
RMP (W·kg⁻¹)	3.26	20.05	11.52	Strong interaction
FPC (N·kg⁻¹)	0.42	2.49	6.48	Moderate interaction
AF (N)	< 3	< 3	< 1	No interaction
AP (W)	< 3	< 3	< 1	No interaction
AFPO (m·s⁻²)	< 0.35	< 0.35	0.36	No interaction
ASPO (m·s⁻²)	0.84	12.86	590.17	Extreme interaction
EDP (N/s)	1.085	0.293	0.818	Weak evidence

Variables include *JHFT* jump height derived from flight time, *JHTOV* Jump height derived from take-off velocity, *VTOV* Vertical take-off velocity, *RMP* Relative maximal power, *FPC* Peak concentric force, *AF* Average force, *AP *Average power, *AFPO* Acceleration during the first and second *ASPO* Halves of the push-off phase

Evidence strength was interpreted according to conventional thresholds (BF₁₀ or BF_incl_: 1–3 = anecdotal, 3–10moderate, 10–30 = strong, 30–100 = very strong, > 100extreme)

**Fig. 2:**
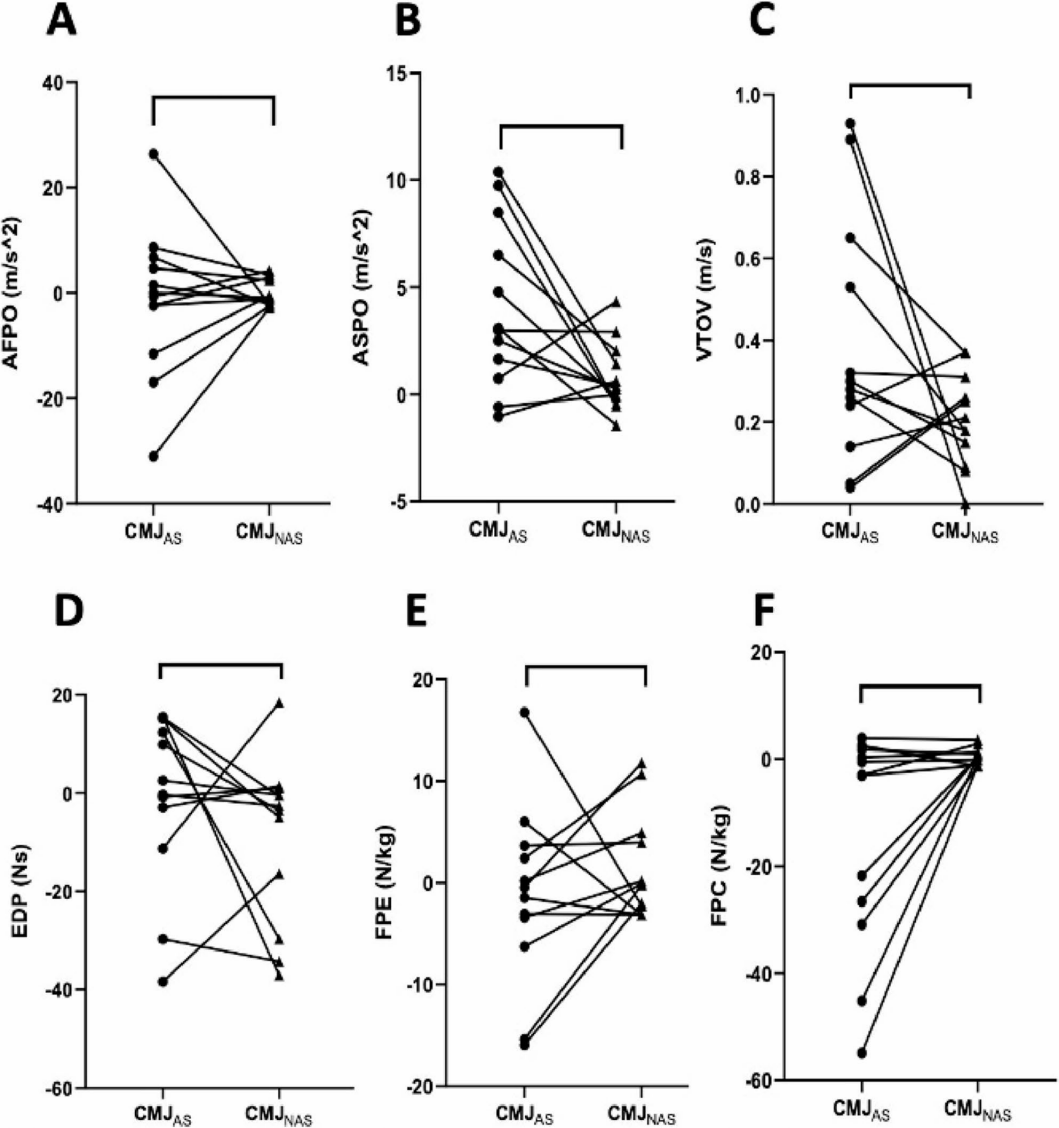
Panels depict within-participant differences for (**A**) acceleration during the first half of the push-off phase (AFPO; CMJ_AS_ BF₁₀ = 0.29, CMJ_NAS_ BF₁₀ = 0.30), (**B**) acceleration during the second half of the push-off phase (ASPO; CMJ_AS_ BF₁₀ = 0.84, CMJ_NAS_ BF₁₀ = 12.86), (**C**) vertical take-off velocity (VTOV; CMJ_AS_ BF₁₀ = 369.74, CMJ_NAS_ BF₁₀ = 40.45), (**D**) eccentric deceleration phase impulse (EDP; weak evidence across conditions), (**E**) force at peak eccentric (FPE; weak evidence across conditions), and (**F**) peak concentric force (FPC; CMJ_AS_ BF₁₀ = 0.42, CMJ_NAS_ BF₁₀ = 2.49). Each line represents an individual participant, with dots indicating condition-specific values

Within-condition Bayesian paired-samples t-test results for motor output variables are illustrated in Fig. 2. JHFT demonstrated extreme evidence for pre–post change in the CMJ_AS_(BF₁₀ = 219.89) and very strong evidence in the CMJ_NAS_(BF₁₀ = 17.48). JHTOV showed strong evidence for change in CMJ_NAS_ (BF₁₀ = 6.61), whereas only anecdotal evidence was observed in CMJ_AS_ (BF₁₀ = 1.09). VTOV exhibited extreme evidence for enhancement in both protocols (CMJ_AS_ BF₁₀ = 369.74; CMJ_NAS_ BF₁₀ = 40.45), indicating robust within-condition changes in take-off mechanics across jump conditions.

**Fig. 3:**
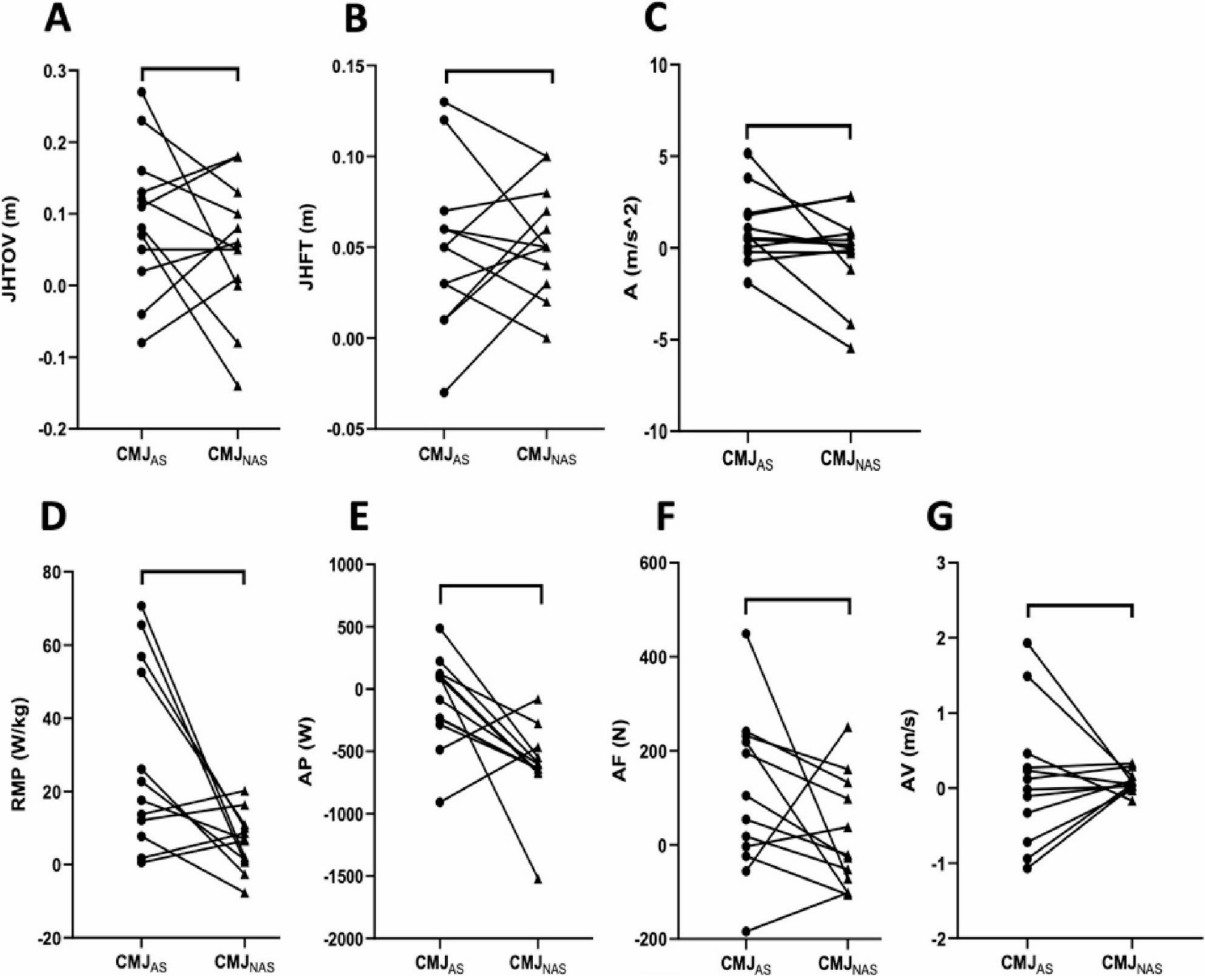
Panels depict within-participant differences between CMJ_AS_ and CMJ_NAS_ for performance-related variables. **A** jump height derived from take-off velocity (JHTOV; CMJ_AS_ BF₁₀ = 1.09, CMJ_NAS_ BF₁₀ = 6.61), (**B**) jump height derived from flight time (JHFT; CMJ_AS_ BF₁₀ = 219.89, CMJ_NAS_ BF₁₀ = 17.48), (**C**) mean acceleration during push-off (A; weak evidence across conditions), (**D**) relative maximal power (RMP; CMJ_AS_ BF₁₀ = 3.26, CMJ_NAS_ BF₁₀ = 20.05), (**E**) average power (AP; weak evidence across conditions), (**F**) average force (AF; weak evidence across conditions), and (**G**) average velocity (AV; weak evidence across conditions). Each line represents an individual participant, with dots indicating condition-specific values

Bayesian paired-samples t-test results for force–power variables are presented in Fig. 3. RMP showed strong evidence for pre–post change in CMJ_NAS_ (BF₁₀ = 20.05), while CMJ_AS_ demonstrated moderate evidence (BF₁₀ = 3.26). FPC exhibited anecdotal-to-moderate evidence in CMJ_NAS_ (BF₁₀ = 2.49) and anecdotal evidence in CMJ_AS_ (BF₁₀ = 0.42). AF and AP did not demonstrate meaningful evidence for change in either condition (BF₁₀ < 3). ASPO demonstrated strong evidence for pre–post change in CMJ_NAS_ (BF₁₀ = 12.86), whereas CMJA_S_ showed only anecdotal evidence (BF₁₀ = 0.84). AFPO did not demonstrate meaningful evidence for change in either protocol (CMJ_AS_ BF₁₀ = 0.29; CMJ_NAS_ BF₁₀ = 0.30). Across functional domains, within-condition Bayesian analyses indicated that motor output variables and late-phase push-off acceleration were the most sensitive to pre–post changes, whereas early-phase acceleration and mean-level force–power outputs showed limited evidence for modulation.

## Discussion

The present study examined protocol-specific neuromuscular responses to acute fatigue during CMJ performance by contrasting jumps performed CMJ_AS_ and CMJ_NAS_. By integrating Bayesian inference with detailed biomechanical and phase-specific analyses, the findings provide novel insight into how upper-limb contribution modulates force–velocity characteristics, SSC behavior, and motor output under fatigued conditions. Beyond traditional performance indices such as jump height, this investigation adopted a system-based perspective that captured both global and phase-specific neuromechanical adaptations, thereby enabling a more nuanced interpretation of fatigue-related changes in CMJ execution. The use of CMJ_AS_ and CMJ_NAS_ as complementary protocols allowed differentiation between whole-body coordination strategies and lower-limb–specific mechanical responses, highlighting how upper-extremity involvement may compensate for or obscure fatigue-induced alterations in lower-limb neuromuscular function. Importantly, the application of Bayesian statistical inference permitted quantification of evidential strength for both the presence and absence of fatigue effects across multiple biomechanical domains. This approach extends prior CMJ research by moving beyond dichotomous significance testing and offering a transparent framework for interpreting protocol-dependent adaptations in force production, velocity generation, and SSC utilization. Collectively, the present findings contribute to a more refined understanding of how acute fatigue reshapes jump mechanics and underscore the importance of protocol selection when CMJ is used for performance monitoring or neuromuscular assessment.

The most robust and consistent findings were observed in global motor output variables, particularly jump height and vertical take-off velocity. Both CMJ protocols demonstrated strong to extreme within-condition evidence for changes in JHFT and VTOV. These outcomes reinforce the fundamental role of eccentric–concentric coupling in CMJ performance, as increased muscle activity during the eccentric phase has been shown to contribute substantially to propulsion during the concentric phase, with eccentric forces reaching 20–60% greater magnitudes than concentric actions. In CMJ_AS_, the involvement of the upper body further amplifies hip joint torque and whole-body momentum transfer, which may increase pushing strength and take-off velocity [13]. However, even minimal disruptions in take-off timing can produce measurable reductions in jump height and velocity, as demonstrated by McMahon et al. [5]. In this context, the present findings support the validity of JHFT, particularly in CMJ_AS_, despite the impulse–momentum approach often being regarded as the gold standard. This aligns with prior literature indicating that higher muscle activity (especially during the eccentric phase) and more efficient SSC utilization are associated with superior vertical displacement and force production [14, 19].

Force–power variables revealed clear protocol-dependent adaptations following fatigue. RMP and FPC showed stronger and more consistent evidence for change under CMJ_NAS_ compared with CMJ_AS_. This suggests that restricting arm swing increases the mechanical and neuromuscular demands placed on the lower extremities, thereby exposing fatigue-related alterations in concentric force–power production more clearly. In contrast, average force and average power remained largely unchanged across both protocols. The absence of meaningful changes in these mean-level metrics suggests that averaged outputs may be less sensitive to short-term neuromuscular alterations induced by acute fatigue. Because these variables represent integrated outputs across the entire movement phase, transient fluctuations in force production may be attenuated when averaged over time. In contrast, peak and phase-specific variables capture instantaneous mechanical outputs during critical phases of the stretch–shortening cycle, making them potentially more sensitive indicators of fatigue-related changes in neuromuscular function.

One of the most novel findings of this study was the divergent response observed in segmental acceleration profiles, particularly during the push-off phase. ASPO demonstrated strong post-fatigue changes in CMJ_NAS_, whereas acceleration during the AFPO remained unchanged across both jump protocols. This pattern suggests a phase-specific neuromuscular adaptation, whereby force production is redistributed toward the later portion of the concentric phase as a compensatory strategy under fatigue. Such delayed force application is consistent with previous evidence showing fatigue-induced shifts in force–time curve peaks and delayed neuromuscular activation [6, 21]. The absence of change in AFPO may indicate that early concentric force transmission is either less adaptable or more susceptible to prior eccentric impairment, potentially due to stretch-reflex suppression, delayed motor unit recruitment, or impaired intermuscular coordination [3, 14]. These findings emphasize the importance of examining acceleration-based parameters in a phase-disaggregated manner. In particular, ASPO appears to be a sensitive biomechanical marker of fatigue-related compensation, especially when upper-limb contributions are minimized.

The presence of a PAP-like response exclusively in CMJ_NAS_, alongside its absence in CMJ_AS_, provides important insight into the interaction between acute fatigue and neuromuscular potentiation. In the absence of upper-limb contribution, CMJ_NAS_ appears to expose fatigue–potentiation dynamics at the level of the lower extremities more directly. The strong Bayesian evidence observed for ASPO in CMJ_NAS_ suggests that acute high-intensity cycling may enhance force application during the late push-off phase, potentially via increased motor unit recruitment efficiency or altered intermuscular coordination following fatigue. In contrast, the inclusion of arm swing in CMJ_AS_ likely redistributes mechanical output across segments, elevating overall performance but simultaneously masking subtle potentiation-related adaptations within the lower limbs. This segmental redistribution may attenuate the detectability of PAP-like responses in CMJ_AS_, despite similar neuromuscular stimuli being applied. Collectively, these findings indicate that CMJ_NAS_ offers greater sensitivity for detecting phase-specific fatigue–potentiation interactions, particularly during the propulsive phase of the jump [25].

Eccentric braking metrics, including FPE and EDP, exhibited only anecdotal changes following fatigue. This observation is consistent with literature suggesting that eccentric force capabilities may be preserved during early stages of fatigue, or that their disruption emerges under repeated-bout or prolonged loading conditions [20, 26]. In the present study, participants may have adopted adaptive movement strategies (such as deeper countermovements or altered joint kinematics) to preserve eccentric output despite neuromuscular decline. This highlights a limitation in interpreting eccentric force alterations without concurrent joint-level kinematic data and underscores the need for future studies integrating kinetic and kinematic analyses.

Collectively, these findings demonstrate that CMJ_AS_ and CMJ_NAS_ provide complementary but distinct information regarding neuromuscular performance. CMJ_AS_ appears to preserve global performance outcomes through whole-body coordination and upper-limb compensation, making it suitable for evaluating overall athletic performance and long-term adaptations. In contrast, CMJ_NAS_ consistently exposed fatigue-related alterations in lower-limb force–power production and late-phase acceleration mechanics, indicating superior sensitivity for detecting acute neuromuscular fatigue.

Previous research has shown that fatigue-related changes in vertical jump biomechanics (such as reduced concentric velocity, altered coordination, and delayed force application) can vary substantially depending on protocol structure and training background [21, 27]. The present findings align with this literature and extend it by demonstrating that CMJ_NAS_ more clearly reflects how neuromotor systems redistribute mechanical workload across movement phases under acute stress. Particularly during the propulsive phase of the jump, when upper-limb contributions are minimized.

A limitation of the present study is that the sample consisted exclusively of male participants. Consequently, the findings should be interpreted with caution when attempting to generalize them to female athletes or to populations with different training backgrounds, competitive levels, or physiological characteristics. Sex-related differences in neuromuscular function, muscle–tendon stiffness, and fatigue responses may influence jump mechanics and stretch–shortening cycle behavior. Therefore, future research including both male and female athletes, as well as participants with diverse training profiles, would be valuable for determining the extent to which the present findings can be generalized across populations.

## Conclusion

This study demonstrates that both CMJ_AS_ and CMJ_NAS_ provide valuable insight into neuromuscular performance, although their sensitivity to acute muscular fatigue differs substantially. Bayesian evidence indicates that CMJ_NAS_ is more responsive to fatigue-related alterations in key biomechanical variables, including take-off velocity–derived jump height, late push-off acceleration, and peak concentric force. By minimizing compensatory upper-limb contributions, CMJ_NAS_ enables a more isolated and interpretable assessment of lower-extremity neuromuscular function under fatigued conditions, whereas CMJ_AS_ remains effective for evaluating whole-body coordination and global performance capacity. Accordingly, CMJ_NAS_ may be more sensitive to detecting fatigue–potentiation interactions following high-intensity cycling exercise, while CMJ_AS_ may mask such responses due to upper-limb contribution. Future studies should extend these findings across different populations, fatigue paradigms, and longitudinal designs to further clarify the diagnostic utility of CMJ-derived metrics.

## Data Availability

The datasets generated and/or analyzed during the current study are available from the corresponding author upon reasonable request.

## Abbreviations

JHTOV (m): The height of the jump calculated from the Take Off velocity as calculated from the force impulse
JHFT (m): The height of jump calculated from the Flight Time
RMP (W/kg-1): Maximal force during the Push Off divided by the body weight
AFPO (m/s^2): Average acceleration between the Lowest Position and the half of the Push Off
ASPO (m/s^2): Average acceleration between the half of the Push Off and the Take Off
VTOV (m/s): Velocity of the vertical movement at the Take Off
AP (W): Average Power
AF (N): Average Force
EDP (Ns): Downward Deceleration Phase Force
FPC (N/kg): Force at Peak Upward movement
